# Crystal structure of *N*′′-benzyl-*N*′′-[3-(benzyl­dimethyl­aza­nium­yl)prop­yl]-*N*,*N*,*N*′,*N*′-tetra­methyl­guanidinium bis­(tetra­phenyl­borate)

**DOI:** 10.1107/S2056989015024639

**Published:** 2015-12-31

**Authors:** Ioannis Tiritiris, Willi Kantlehner

**Affiliations:** a Fakultät Chemie/Organische Chemie, Hochschule Aalen, Beethovenstrasse 1, D-73430 Aalen, Germany

**Keywords:** crystal structure, guanidinium salt, tetra­phenyl­borate, C—H⋯π inter­actions

## Abstract

In the crystal structure of the title salt, C_24_H_38_N_4_^2+^·2C_24_H_20_B^−^, the C—N bond lengths in the central CN_3_ unit of the guanidinium ion are 1.3364 (13), 1.3407 (13) and 1.3539 (13) Å, indicating partial double-bond character. The central C atom is bonded to the three N atoms in a nearly ideal trigonal–planar geometry and the positive charge is delocalized in the CN_3_ plane. The bonds between the N atoms and the terminal methyl groups of the guanidinium moiety and the four C—N bonds to the central N atom of the (benzyl­dimethyl­aza­nium­yl)propyl group have single-bond character. In the crystal, C—H⋯π inter­actions between the guanidin­ium H atoms and the phenyl C atoms of the tetra­phenyl­borate ions are present, leading to the formation of a two-dimensional supra­molecular pattern parallel to the *ac* plane.

## Related literature   

For the crystal structures of alkali metal tetra­phenyl­borates, see: Behrens *et al.* (2012*a* ▸). For the synthesis of *N*′′-[3-(di­methyl­amino)­prop­yl]-*N*,*N*,*N*′,*N*′-tetra­methyl­guanidine, see: Tiritiris & Kantlehner (2012*b* ▸). For the crystal structure of *N*,*N*,*N*′,*N*′,*N*′′-penta­methyl-*N*′′-[3-(tri­methyl­aza­nium­yl)propyl]guanidinium bis­(tetra­phenyl­borate), see: Tiritiris (2013*a* ▸). For the crystal structure of *N*-[3-(benzyl­dimethyl­aza­niumyl)prop­yl]-*N*′,*N*′,*N*′′,*N*′′-tetra­methyl­guanidinium bis­(tetraphenyl­borate), see: Tiritiris (2013*b* ▸).
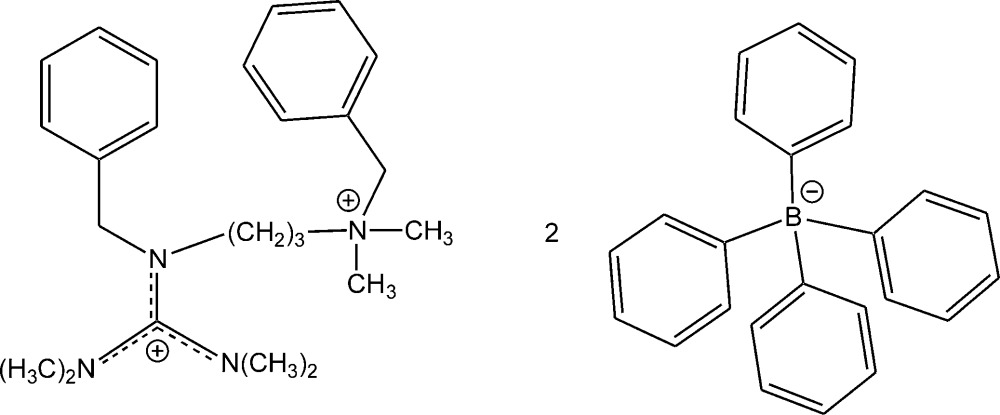


## Experimental   

### Crystal data  

C_24_H_38_N_4_^2+^·2C_24_H_20_B^−^
*M* *_r_* = 1021.00
Monoclinic, 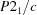

*a* = 20.3677 (9) Å
*b* = 12.1101 (5) Å
*c* = 25.5580 (12) Å
β = 112.507 (2)°
*V* = 5823.9 (5) Å^3^
*Z* = 4
Mo *K*α radiation
μ = 0.07 mm^−1^
*T* = 100 K
0.38 × 0.25 × 0.12 mm

### Data collection  

Bruker Kappa APEXII DUO diffractometer
70711 measured reflections
17795 independent reflections
13809 reflections with *I* > 2σ(*I*)
*R*_int_ = 0.032

### Refinement  

*R*[*F* ^2^ > 2σ(*F* ^2^)] = 0.043
*wR*(*F* ^2^) = 0.117
*S* = 1.03
17795 reflections
709 parameters
H-atom parameters constrained
Δρ_max_ = 0.38 e Å^−3^
Δρ_min_ = −0.31 e Å^−3^

### 

Data collection: *APEX2* (Bruker, 2008 ▸); cell refinement: *SAINT* (Bruker, 2008 ▸); data reduction: *SAINT*; program(s) used to solve structure: *SHELXS97* (Sheldrick, 2008 ▸); program(s) used to refine structure: *SHELXL2014* (Sheldrick, 2015 ▸); molecular graphics: *DIAMOND* (Brandenburg & Putz, 2005 ▸); software used to prepare material for publication: *SHELXL2014*.

## Supplementary Material

Crystal structure: contains datablock(s) I, global. DOI: 10.1107/S2056989015024639/rz5181sup1.cif

Structure factors: contains datablock(s) I. DOI: 10.1107/S2056989015024639/rz5181Isup2.hkl

Supporting information file. DOI: 10.1107/S2056989015024639/rz5181Isup3.cml

**Fig. 1.**
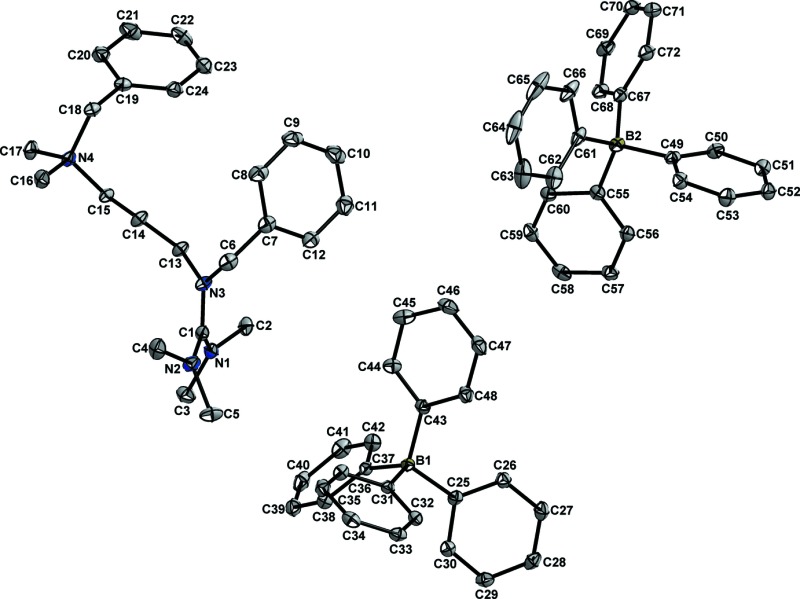
The structure of the title compound with displacement ellipsoids drawn at the 50% probability level. Hydrogen atoms are omitted for clarity.

**Fig. 2.**
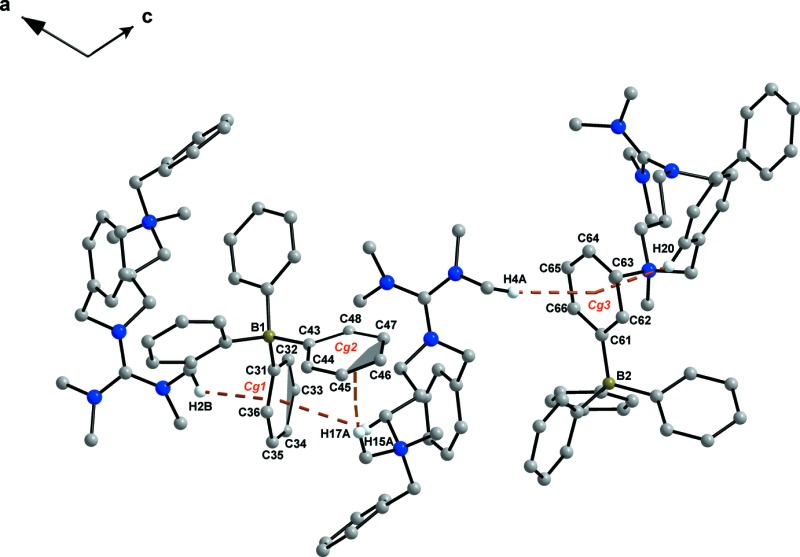
C—H···π interactions (brown dashed lines) between the hydrogen atoms of the guanidinium ion and the phenyl rings (centroids) of the tetraphenylborate ions in the crystal structure of the title compound (view perpendicular to the ac plane). Hydrogen atoms not involved in hydrogen bonding are omitted.

. DOI: 10.1107/S2056989015024639/rz5181fig1.tif
The structure of the title compound with displacement ellipsoids drawn at the 50% probability level. Hydrogen atoms are omitted for clarity.

ac . DOI: 10.1107/S2056989015024639/rz5181fig2.tif
C—H⋯π inter­actions (brown dashed lines) between the hydrogen atoms of the guanidinium ion and the phenyl rings (centroids) of the tetra­phenyl­borate ions in the crystal structure of the title compound (view perpendicular to the *ac* plane). Hydrogen atoms not involved in hydrogen bonding are omitted.

CCDC reference: 1443781

Additional supporting information:  crystallographic information; 3D view; checkCIF report

## Figures and Tables

**Table 1:** Hydrogen-bond geometry (Å, °) *Cg*1, *Cg*2 and *Cg*3 are the centroids of the C31–C36, C43–C48 and C61–C66 aromatic rings, respectively.

*D*—H⋯*A*	*D*—H	H⋯*A*	*D*⋯*A*	*D*—H⋯*A*
C2—H2*B*⋯*Cg*1^i^	0.98	2.76	3.441	127
C15—H15*A*⋯*Cg*1^ii^	0.99	2.94	3.538	120
C17—H17*A*⋯*Cg*2^ii^	0.98	2.76	3.716	166
C4—H4*A*⋯*Cg*3^iii^	0.98	2.88	3.840	169
C20—H20⋯*Cg*3^iv^	0.95	2.91	3.719	144

Symmetry codes: (i) 
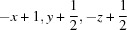
; (ii) 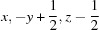; (iii) 
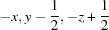
; (iv) 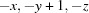.

## supplementary crystallographic information

### S1. Comment

The synthesis of *N*''-[3-(dimethylamino)propyl]-*N*,*N*,*N*',*N*'-tetramethylguanidine is well known in the literature (Tiritiris & Kantlehner, 2012*b*). Electrophiles can attack at both, on the imine nitrogen of the guanidine function, as well as on the nitrogen atom of the (dimethylamino)propyl group. By alkylation with two equivalents of dimethyl sulfate, a permethylated waxy guanidinium methyl sulfate salt was obtained. After anion exchange with sodium tetraphenylborate, crystalline *N*,*N*,*N*',*N*',*N*''-pentamethyl-*N*''-[3-(trimethylazaniumyl)propyl]guanidinium bis(tetraphenylborate) emerged, which has been structurally characterized (Tiritiris, 2013*a*). By alkylation with benzyl bromide and subsequent anion exchange, the here presented title salt is the second one in our serie, whose crystal structure has been determined. Prominent bond parameters in the guanidinium ion are: C1–N1 = 1.3407 (13) Å, C1–N2 = 1.3364 (13) Å and C1–N3 = 1.3539 (13) Å, indicating partial double-bond character. The N–C1–N angles are: 121.16 (9)° (N1–C1–N2), 119.26 (9)° (N2–C1–N3) and 119.57 (9)° (N1–C1–N3), which indicates a nearly ideal trigonal-planar surrounding of the carbon centre by the nitrogen atoms. The positive charge is completely delocalized on the CN_3_ plane (Fig. 1). The bonds between the N atoms and the terminal *C*-methyl groups of the guanidinium moiety all have values close to a typical single bond [1.4639 (13)–1.4673 (14) Å]. The C–N bond lengths in the terminal benzyldimethylammonium group are slightly elongated [1.4994 (13)–1.5293 (13) Å]. The bond lengths and angles in the tetraphenylborate ions are in good agreement with the data from the crystal structure analysis of the alkali metal tetraphenylborates (Behrens *et al.*, 2012*a*). C–H···π interactions between the hydrogen atoms of –N(CH_3_)_2_, –CH_2_ and benzyl groups of the guanidinium ion and the phenyl carbon atoms (centroids: *Cg*1 = C31–C36, *Cg*2 = C43–C48 and *Cg*3 = C61–C66) of the tetraphenylborate ions are present, ranging from 2.76 (2) to 2.94 (2) Å (Fig. 2; Table 1). This leads to the formation of a two- dimensional supramolecular pattern along the **ac** plane. Such type of C–H···π interactions have been also observed in *N*-[3-(benzyldimethylazaniumyl)propyl]- *N*',*N*',*N*'',*N*''-tetramethylguanidinium bis(tetraphenylborate) (Tiritiris, 2013*b*).

### S2. Experimental

The title compound was obtained by reaction of *N*''-[3-(dimethylamino)propyl]-*N*,*N*,*N*',*N*'-tetramethylguanidine (Tiritiris & Kantlehner, 2012*b*) with two equivalents benzyl bromide in acetonitrile. After evaporation of the solvent the crude *N*''-benzyl-*N*''-[3-(benzyldimethylazaniumyl)propyl]-*N*,*N*,*N*',*N*'-tetramethylguanidinium dibromide (I) was washed with diethylether and dried *in vacuo*. 1.52 g (2.8 mmol) of (I) was dissolved in 20 ml acetonitrile and 1.92 g (5.6 mmol) of sodium tetraphenylborate in 20 ml acetonitrile was added. After stirring for one hour at room temperature, the precipitated sodium bromide was filtered off. The title compound crystallized from a saturated acetonitrile solution after several weeks at 273 K, forming colorless single crystals. Yield: 2.20 g (77%).

### S3. Refinement

The hydrogen atoms of the methyl groups were allowed to rotate with a fixed angle around the C–N bond to best fit the experimental electron density, with *U*_iso_(H) set to 1.5*U*_eq_(C) and d(C—H) = 0.98 Å. The remaining H atoms were placed in calculated positions with d(C—H) = 0.99 Å (H atoms in CH_2_ groups) and (C—H) = 0.95 Å (H atoms in aromatic rings). They were refined using a riding model, with *U*_iso_(H) set to 1.2 *U*_eq_(C).

### Crystal data

**Table:**

C_24_H_38_N_4_^2+^·2C_24_H_20_B^−^	*F*(000) = 2192
*M**_r_* = 1021.00	*D*_x_ = 1.164 Mg m^−^^3^
Monoclinic, *P*2_1_/*c*	Mo *K*α radiation, λ = 0.71073 Å
*a* = 20.3677 (9) Å	Cell parameters from 70711 reflections
*b* = 12.1101 (5) Å	θ = 1.7–30.7°
*c* = 25.5580 (12) Å	µ = 0.07 mm^−^^1^
β = 112.507 (2)°	*T* = 100 K
*V* = 5823.9 (5) Å^3^	Block, colorless
*Z* = 4	0.38 × 0.25 × 0.12 mm

### Data collection

**Table:**

Bruker Kappa APEXII DUO diffractometer	13809 reflections with *I* > 2σ(*I*)
Radiation source: fine-focus sealed tube	*R*_int_ = 0.032
Triumph monochromator	θ_max_ = 30.7°, θ_min_ = 1.7°
φ scans, and ω scans	*h* = −29→28
70711 measured reflections	*k* = −17→15
17795 independent reflections	*l* = −36→36

### Refinement

**Table:**

Refinement on *F*^2^	Primary atom site location: structure-invariant direct methods
Least-squares matrix: full	Secondary atom site location: difference Fourier map
*R*[*F*^2^ > 2σ(*F*^2^)] = 0.043	Hydrogen site location: inferred from neighbouring sites
*wR*(*F*^2^) = 0.117	H-atom parameters constrained
*S* = 1.03	*w* = 1/[σ^2^(*F*_o_^2^) + (0.0586*P*)^2^ + 1.3509*P*] where *P* = (*F*_o_^2^ + 2*F*_c_^2^)/3
17795 reflections	(Δ/σ)_max_ < 0.001
709 parameters	Δρ_max_ = 0.38 e Å^−^^3^
0 restraints	Δρ_min_ = −0.31 e Å^−^^3^

### Special details

**Table:**

Geometry. All e.s.d.'s (except the e.s.d. in the dihedral angle between two l.s. planes) are estimated using the full covariance matrix. The cell e.s.d.'s are taken into account individually in the estimation of e.s.d.'s in distances, angles and torsion angles; correlations between e.s.d.'s in cell parameters are only used when they are defined by crystal symmetry. An approximate (isotropic) treatment of cell e.s.d.'s is used for estimating e.s.d.'s involving l.s. planes.
Refinement. Refinement of *F*^2^ against ALL reflections. The weighted *R*-factor *wR* and goodness of fit *S* are based on *F*^2^, conventional *R*-factors *R* are based on *F*, with *F* set to zero for negative *F*^2^. The threshold expression of *F*^2^ > σ(*F*^2^) is used only for calculating *R*-factors(gt) *etc*. and is not relevant to the choice of reflections for refinement. *R*-factors based on *F*^2^ are statistically about twice as large as those based on *F*, and *R*- factors based on ALL data will be even larger. The crystal was refined as a 2-component inversion twin.

### Fractional atomic coordinates and isotropic or equivalent isotropic displacement parameters (Å^2^)

**Table:**

	*x*	*y*	*z*	*U*_iso_*/*U*_eq_	
N1	0.42988 (5)	0.26256 (7)	0.07644 (4)	0.01750 (16)	
N2	0.35454 (5)	0.13022 (7)	0.08976 (4)	0.01989 (17)	
N3	0.30742 (4)	0.29028 (7)	0.03943 (3)	0.01659 (16)	
N4	0.17834 (4)	0.31337 (7)	−0.17076 (3)	0.01554 (16)	
C1	0.36450 (5)	0.22714 (8)	0.06888 (4)	0.01603 (18)	
C2	0.44809 (6)	0.38008 (9)	0.08200 (4)	0.0197 (2)	
H2A	0.4547	0.4054	0.0479	0.030*	
H2B	0.4922	0.3913	0.1152	0.030*	
H2C	0.4096	0.4222	0.0867	0.030*	
C3	0.48710 (6)	0.18814 (10)	0.07763 (5)	0.0245 (2)	
H3A	0.5228	0.1835	0.1163	0.037*	
H3B	0.5093	0.2166	0.0524	0.037*	
H3C	0.4675	0.1145	0.0649	0.037*	
C4	0.29137 (6)	0.06255 (9)	0.06026 (5)	0.0242 (2)	
H4A	0.2597	0.0651	0.0810	0.036*	
H4B	0.3058	−0.0140	0.0582	0.036*	
H4C	0.2664	0.0913	0.0219	0.036*	
C5	0.40727 (6)	0.08275 (10)	0.14151 (5)	0.0279 (2)	
H5A	0.4338	0.0245	0.1315	0.042*	
H5B	0.3831	0.0514	0.1647	0.042*	
H5C	0.4402	0.1406	0.1630	0.042*	
C6	0.24849 (5)	0.30775 (9)	0.05902 (4)	0.01947 (19)	
H6A	0.2600	0.2719	0.0963	0.023*	
H6B	0.2044	0.2740	0.0318	0.023*	
C7	0.23730 (5)	0.43013 (9)	0.06391 (4)	0.0193 (2)	
C8	0.17815 (6)	0.48332 (10)	0.02458 (5)	0.0230 (2)	
H8	0.1430	0.4416	−0.0041	0.028*	
C9	0.17034 (6)	0.59713 (10)	0.02714 (5)	0.0276 (2)	
H9	0.1303	0.6329	−0.0001	0.033*	
C10	0.22059 (7)	0.65826 (10)	0.06926 (5)	0.0290 (2)	
H10	0.2152	0.7360	0.0707	0.035*	
C11	0.27903 (6)	0.60616 (10)	0.10949 (5)	0.0269 (2)	
H11	0.3133	0.6480	0.1387	0.032*	
C12	0.28713 (6)	0.49265 (10)	0.10673 (5)	0.0224 (2)	
H12	0.3270	0.4572	0.1343	0.027*	
C13	0.30239 (5)	0.34444 (8)	−0.01334 (4)	0.01673 (18)	
H13A	0.3456	0.3279	−0.0209	0.020*	
H13B	0.2996	0.4254	−0.0093	0.020*	
C14	0.23657 (5)	0.30416 (9)	−0.06305 (4)	0.01831 (19)	
H14A	0.2318	0.2232	−0.0607	0.022*	
H14B	0.1934	0.3393	−0.0616	0.022*	
C15	0.24436 (5)	0.33405 (8)	−0.11817 (4)	0.01549 (17)	
H15A	0.2571	0.4132	−0.1170	0.019*	
H15B	0.2841	0.2908	−0.1210	0.019*	
C16	0.14732 (6)	0.20164 (8)	−0.16939 (4)	0.0202 (2)	
H16A	0.1087	0.1869	−0.2059	0.030*	
H16B	0.1287	0.1997	−0.1393	0.030*	
H16C	0.1843	0.1453	−0.1621	0.030*	
C17	0.20009 (6)	0.31652 (9)	−0.22064 (4)	0.0203 (2)	
H17A	0.2340	0.2569	−0.2172	0.031*	
H17B	0.2224	0.3878	−0.2216	0.031*	
H17C	0.1581	0.3069	−0.2556	0.031*	
C18	0.12001 (5)	0.39863 (8)	−0.17835 (4)	0.01773 (19)	
H18A	0.0798	0.3836	−0.2146	0.021*	
H18B	0.1024	0.3888	−0.1475	0.021*	
C19	0.14251 (5)	0.51683 (8)	−0.17835 (4)	0.01772 (19)	
C20	0.14317 (6)	0.56721 (9)	−0.22727 (5)	0.0229 (2)	
H20	0.1303	0.5263	−0.2615	0.027*	
C21	0.16279 (7)	0.67763 (10)	−0.22577 (5)	0.0292 (3)	
H21	0.1642	0.7116	−0.2588	0.035*	
C22	0.18025 (7)	0.73815 (9)	−0.17629 (6)	0.0282 (2)	
H22	0.1936	0.8135	−0.1756	0.034*	
C23	0.17838 (6)	0.68936 (9)	−0.12783 (5)	0.0247 (2)	
H23	0.1897	0.7313	−0.0941	0.030*	
C24	0.15983 (6)	0.57893 (9)	−0.12892 (5)	0.0203 (2)	
H24	0.1589	0.5452	−0.0956	0.024*	
B1	0.46620 (6)	0.23547 (9)	0.36054 (4)	0.01377 (19)	
C25	0.53180 (5)	0.27524 (8)	0.41906 (4)	0.01506 (17)	
C26	0.53687 (5)	0.37636 (8)	0.44722 (4)	0.01841 (19)	
H26	0.4994	0.4284	0.4321	0.022*	
C27	0.59477 (6)	0.40360 (9)	0.49663 (5)	0.0222 (2)	
H27	0.5956	0.4725	0.5147	0.027*	
C28	0.65101 (6)	0.33058 (9)	0.51945 (4)	0.0217 (2)	
H28	0.6902	0.3484	0.5532	0.026*	
C29	0.64893 (6)	0.23066 (9)	0.49194 (4)	0.0208 (2)	
H29	0.6874	0.1803	0.5065	0.025*	
C30	0.59059 (5)	0.20459 (8)	0.44326 (4)	0.01846 (19)	
H30	0.5903	0.1357	0.4254	0.022*	
C31	0.43022 (5)	0.12063 (8)	0.37144 (4)	0.01409 (17)	
C32	0.43723 (5)	0.08192 (8)	0.42503 (4)	0.01565 (18)	
H32	0.4664	0.1220	0.4576	0.019*	
C33	0.40298 (5)	−0.01320 (8)	0.43244 (4)	0.01751 (18)	
H33	0.4094	−0.0366	0.4696	0.021*	
C34	0.35964 (5)	−0.07381 (8)	0.38589 (5)	0.01895 (19)	
H34	0.3368	−0.1391	0.3908	0.023*	
C35	0.35032 (5)	−0.03701 (9)	0.33182 (5)	0.01950 (19)	
H35	0.3204	−0.0767	0.2994	0.023*	
C36	0.38493 (5)	0.05798 (8)	0.32539 (4)	0.01735 (18)	
H36	0.3776	0.0817	0.2881	0.021*	
C37	0.50326 (5)	0.21940 (8)	0.31399 (4)	0.01491 (17)	
C38	0.52380 (5)	0.11719 (9)	0.29919 (4)	0.01828 (19)	
H38	0.5107	0.0516	0.3131	0.022*	
C39	0.56287 (6)	0.10823 (10)	0.26469 (5)	0.0229 (2)	
H39	0.5759	0.0374	0.2559	0.027*	
C40	0.58261 (6)	0.20214 (10)	0.24331 (5)	0.0250 (2)	
H40	0.6096	0.1963	0.2202	0.030*	
C41	0.56232 (6)	0.30497 (10)	0.25618 (5)	0.0240 (2)	
H41	0.5748	0.3701	0.2414	0.029*	
C42	0.52364 (6)	0.31235 (9)	0.29076 (4)	0.01938 (19)	
H42	0.5104	0.3835	0.2990	0.023*	
C43	0.40014 (5)	0.32412 (8)	0.33856 (4)	0.01539 (17)	
C44	0.36416 (5)	0.35274 (9)	0.28117 (4)	0.01945 (19)	
H44	0.3797	0.3210	0.2539	0.023*	
C45	0.30691 (6)	0.42552 (10)	0.26273 (5)	0.0246 (2)	
H45	0.2839	0.4416	0.2235	0.030*	
C46	0.28330 (6)	0.47461 (9)	0.30136 (5)	0.0266 (2)	
H46	0.2453	0.5263	0.2891	0.032*	
C47	0.31613 (6)	0.44687 (9)	0.35821 (5)	0.0249 (2)	
H47	0.3003	0.4793	0.3852	0.030*	
C48	0.37217 (5)	0.37170 (9)	0.37589 (4)	0.01895 (19)	
H48	0.3924	0.3517	0.4148	0.023*	
B2	−0.05215 (6)	0.70020 (10)	0.40237 (5)	0.0163 (2)	
C49	−0.04678 (5)	0.73498 (8)	0.46616 (4)	0.01608 (18)	
C50	−0.01343 (5)	0.83323 (9)	0.49223 (4)	0.01913 (19)	
H50	0.0002	0.8850	0.4704	0.023*	
C51	0.00054 (6)	0.85813 (9)	0.54879 (4)	0.0213 (2)	
H51	0.0240	0.9250	0.5649	0.026*	
C52	−0.01996 (6)	0.78472 (9)	0.58155 (4)	0.0218 (2)	
H52	−0.0102	0.8005	0.6202	0.026*	
C53	−0.05484 (6)	0.68795 (9)	0.55698 (4)	0.0221 (2)	
H53	−0.0699	0.6378	0.5787	0.026*	
C54	−0.06784 (5)	0.66418 (9)	0.50041 (4)	0.01887 (19)	
H54	−0.0918	0.5976	0.4845	0.023*	
C55	0.02320 (5)	0.64157 (8)	0.40784 (4)	0.01560 (18)	
C56	0.08370 (5)	0.63716 (8)	0.45817 (4)	0.01707 (18)	
H56	0.0813	0.6677	0.4916	0.020*	
C57	0.14745 (5)	0.58952 (8)	0.46106 (5)	0.0200 (2)	
H57	0.1872	0.5881	0.4961	0.024*	
C58	0.15304 (6)	0.54410 (8)	0.41296 (5)	0.0205 (2)	
H58	0.1965	0.5123	0.4147	0.025*	
C59	0.09406 (6)	0.54589 (8)	0.36225 (5)	0.01945 (19)	
H59	0.0970	0.5152	0.3290	0.023*	
C60	0.03075 (6)	0.59263 (8)	0.36028 (4)	0.01809 (19)	
H60	−0.0092	0.5916	0.3254	0.022*	
C61	−0.11684 (5)	0.60929 (10)	0.37559 (4)	0.0204 (2)	
C62	−0.10414 (6)	0.49566 (10)	0.38699 (5)	0.0259 (2)	
H62	−0.0571	0.4723	0.4090	0.031*	
C63	−0.15750 (7)	0.41580 (12)	0.36737 (6)	0.0349 (3)	
H63	−0.1463	0.3402	0.3764	0.042*	
C64	−0.22645 (8)	0.44651 (14)	0.33485 (5)	0.0412 (4)	
H64	−0.2630	0.3927	0.3209	0.049*	
C65	−0.24121 (7)	0.55746 (15)	0.32296 (5)	0.0392 (4)	
H65	−0.2884	0.5798	0.3006	0.047*	
C66	−0.18766 (6)	0.63718 (12)	0.34342 (4)	0.0269 (2)	
H66	−0.1997	0.7127	0.3352	0.032*	
C67	−0.06546 (5)	0.81251 (9)	0.36295 (4)	0.01731 (18)	
C68	−0.02646 (5)	0.84143 (9)	0.32986 (4)	0.01829 (19)	
H68	0.0094	0.7924	0.3287	0.022*	
C69	−0.03839 (6)	0.93941 (9)	0.29857 (4)	0.0212 (2)	
H69	−0.0110	0.9552	0.2766	0.025*	
C70	−0.08985 (6)	1.01377 (9)	0.29939 (5)	0.0233 (2)	
H70	−0.0981	1.0804	0.2782	0.028*	
C71	−0.12914 (6)	0.98859 (10)	0.33193 (5)	0.0245 (2)	
H71	−0.1644	1.0385	0.3332	0.029*	
C72	−0.11691 (6)	0.89051 (10)	0.36257 (4)	0.0223 (2)	
H72	−0.1447	0.8754	0.3843	0.027*	

### Atomic displacement parameters (Å^2^)

**Table:**

	*U*^11^	*U*^22^	*U*^33^	*U*^12^	*U*^13^	*U*^23^
N1	0.0161 (4)	0.0165 (4)	0.0189 (4)	−0.0008 (3)	0.0056 (3)	−0.0011 (3)
N2	0.0218 (4)	0.0184 (4)	0.0173 (4)	−0.0029 (3)	0.0050 (3)	0.0030 (3)
N3	0.0154 (4)	0.0199 (4)	0.0148 (4)	−0.0009 (3)	0.0061 (3)	0.0032 (3)
N4	0.0169 (4)	0.0160 (4)	0.0138 (4)	−0.0020 (3)	0.0060 (3)	0.0003 (3)
C1	0.0180 (4)	0.0167 (4)	0.0126 (4)	−0.0019 (3)	0.0049 (4)	−0.0008 (3)
C2	0.0184 (5)	0.0185 (5)	0.0214 (5)	−0.0051 (4)	0.0066 (4)	−0.0034 (4)
C3	0.0203 (5)	0.0247 (5)	0.0288 (6)	0.0040 (4)	0.0097 (4)	0.0006 (4)
C4	0.0295 (6)	0.0205 (5)	0.0232 (5)	−0.0092 (4)	0.0107 (4)	−0.0013 (4)
C5	0.0279 (6)	0.0298 (6)	0.0239 (5)	0.0051 (5)	0.0076 (5)	0.0119 (5)
C6	0.0165 (4)	0.0249 (5)	0.0188 (5)	−0.0019 (4)	0.0087 (4)	0.0035 (4)
C7	0.0167 (5)	0.0263 (5)	0.0176 (5)	0.0004 (4)	0.0094 (4)	0.0027 (4)
C8	0.0161 (5)	0.0329 (6)	0.0207 (5)	0.0015 (4)	0.0079 (4)	0.0023 (4)
C9	0.0226 (5)	0.0357 (6)	0.0262 (6)	0.0107 (5)	0.0113 (5)	0.0064 (5)
C10	0.0318 (6)	0.0278 (6)	0.0327 (6)	0.0071 (5)	0.0181 (5)	0.0014 (5)
C11	0.0259 (6)	0.0305 (6)	0.0261 (6)	−0.0003 (5)	0.0118 (5)	−0.0052 (5)
C12	0.0182 (5)	0.0310 (6)	0.0182 (5)	0.0027 (4)	0.0073 (4)	0.0005 (4)
C13	0.0170 (4)	0.0199 (5)	0.0135 (4)	−0.0023 (4)	0.0061 (4)	0.0031 (3)
C14	0.0178 (5)	0.0227 (5)	0.0140 (4)	−0.0037 (4)	0.0057 (4)	0.0030 (4)
C15	0.0148 (4)	0.0175 (4)	0.0139 (4)	−0.0010 (3)	0.0051 (3)	0.0011 (3)
C16	0.0235 (5)	0.0165 (5)	0.0200 (5)	−0.0059 (4)	0.0075 (4)	−0.0007 (4)
C17	0.0251 (5)	0.0235 (5)	0.0153 (4)	−0.0014 (4)	0.0110 (4)	−0.0003 (4)
C18	0.0151 (4)	0.0195 (5)	0.0177 (5)	0.0002 (4)	0.0053 (4)	0.0014 (4)
C19	0.0149 (4)	0.0183 (5)	0.0196 (5)	0.0020 (3)	0.0062 (4)	0.0020 (4)
C20	0.0276 (5)	0.0214 (5)	0.0207 (5)	0.0042 (4)	0.0106 (4)	0.0033 (4)
C21	0.0397 (7)	0.0219 (5)	0.0330 (6)	0.0057 (5)	0.0217 (5)	0.0086 (5)
C22	0.0317 (6)	0.0162 (5)	0.0411 (7)	0.0023 (4)	0.0189 (5)	0.0021 (5)
C23	0.0240 (5)	0.0206 (5)	0.0292 (6)	0.0029 (4)	0.0098 (5)	−0.0037 (4)
C24	0.0195 (5)	0.0211 (5)	0.0206 (5)	0.0035 (4)	0.0080 (4)	0.0016 (4)
B1	0.0150 (5)	0.0129 (4)	0.0134 (5)	0.0005 (4)	0.0055 (4)	0.0003 (4)
C25	0.0168 (4)	0.0152 (4)	0.0141 (4)	−0.0012 (3)	0.0070 (4)	0.0013 (3)
C26	0.0185 (5)	0.0161 (4)	0.0199 (5)	−0.0003 (4)	0.0066 (4)	−0.0008 (4)
C27	0.0236 (5)	0.0189 (5)	0.0223 (5)	−0.0036 (4)	0.0068 (4)	−0.0054 (4)
C28	0.0188 (5)	0.0246 (5)	0.0178 (5)	−0.0052 (4)	0.0027 (4)	−0.0016 (4)
C29	0.0169 (5)	0.0218 (5)	0.0206 (5)	0.0008 (4)	0.0039 (4)	0.0025 (4)
C30	0.0186 (5)	0.0172 (4)	0.0185 (5)	0.0005 (4)	0.0059 (4)	−0.0010 (4)
C31	0.0133 (4)	0.0136 (4)	0.0156 (4)	0.0026 (3)	0.0058 (3)	0.0005 (3)
C32	0.0158 (4)	0.0158 (4)	0.0155 (4)	0.0017 (3)	0.0062 (4)	−0.0005 (3)
C33	0.0177 (4)	0.0174 (4)	0.0196 (5)	0.0031 (4)	0.0095 (4)	0.0037 (4)
C34	0.0166 (4)	0.0151 (4)	0.0273 (5)	0.0001 (4)	0.0108 (4)	0.0006 (4)
C35	0.0162 (4)	0.0199 (5)	0.0215 (5)	−0.0026 (4)	0.0062 (4)	−0.0047 (4)
C36	0.0163 (4)	0.0189 (5)	0.0156 (4)	−0.0004 (4)	0.0048 (4)	0.0000 (4)
C37	0.0137 (4)	0.0171 (4)	0.0125 (4)	−0.0001 (3)	0.0034 (3)	−0.0007 (3)
C38	0.0183 (5)	0.0186 (5)	0.0169 (4)	0.0011 (4)	0.0056 (4)	−0.0009 (4)
C39	0.0200 (5)	0.0282 (5)	0.0195 (5)	0.0030 (4)	0.0065 (4)	−0.0060 (4)
C40	0.0198 (5)	0.0402 (6)	0.0169 (5)	−0.0004 (4)	0.0090 (4)	−0.0028 (4)
C41	0.0231 (5)	0.0303 (6)	0.0206 (5)	−0.0049 (4)	0.0104 (4)	0.0029 (4)
C42	0.0196 (5)	0.0194 (5)	0.0199 (5)	−0.0018 (4)	0.0084 (4)	−0.0003 (4)
C43	0.0153 (4)	0.0129 (4)	0.0178 (4)	−0.0011 (3)	0.0061 (4)	0.0001 (3)
C44	0.0181 (5)	0.0203 (5)	0.0202 (5)	0.0017 (4)	0.0077 (4)	0.0037 (4)
C45	0.0197 (5)	0.0256 (5)	0.0261 (5)	0.0037 (4)	0.0061 (4)	0.0092 (4)
C46	0.0188 (5)	0.0197 (5)	0.0385 (6)	0.0054 (4)	0.0078 (5)	0.0016 (4)
C47	0.0197 (5)	0.0223 (5)	0.0337 (6)	0.0008 (4)	0.0112 (5)	−0.0089 (4)
C48	0.0166 (5)	0.0194 (5)	0.0207 (5)	−0.0012 (4)	0.0069 (4)	−0.0036 (4)
B2	0.0134 (5)	0.0211 (5)	0.0144 (5)	0.0000 (4)	0.0056 (4)	0.0004 (4)
C49	0.0128 (4)	0.0196 (5)	0.0157 (4)	0.0027 (3)	0.0053 (3)	0.0018 (3)
C50	0.0183 (5)	0.0203 (5)	0.0179 (5)	−0.0001 (4)	0.0059 (4)	0.0027 (4)
C51	0.0208 (5)	0.0209 (5)	0.0193 (5)	0.0009 (4)	0.0046 (4)	−0.0017 (4)
C52	0.0231 (5)	0.0265 (5)	0.0165 (5)	0.0045 (4)	0.0084 (4)	−0.0008 (4)
C53	0.0246 (5)	0.0260 (5)	0.0194 (5)	0.0010 (4)	0.0125 (4)	0.0023 (4)
C54	0.0186 (5)	0.0207 (5)	0.0186 (5)	0.0000 (4)	0.0084 (4)	0.0000 (4)
C55	0.0160 (4)	0.0144 (4)	0.0168 (4)	−0.0012 (3)	0.0069 (4)	0.0006 (3)
C56	0.0163 (4)	0.0164 (4)	0.0181 (4)	−0.0003 (3)	0.0061 (4)	0.0001 (3)
C57	0.0161 (5)	0.0175 (5)	0.0237 (5)	0.0005 (4)	0.0044 (4)	0.0005 (4)
C58	0.0179 (5)	0.0149 (4)	0.0310 (6)	0.0017 (4)	0.0119 (4)	0.0012 (4)
C59	0.0238 (5)	0.0152 (4)	0.0229 (5)	−0.0007 (4)	0.0130 (4)	−0.0009 (4)
C60	0.0199 (5)	0.0169 (4)	0.0178 (5)	−0.0009 (4)	0.0075 (4)	0.0003 (4)
C61	0.0178 (5)	0.0314 (6)	0.0132 (4)	−0.0043 (4)	0.0072 (4)	−0.0025 (4)
C62	0.0265 (6)	0.0299 (6)	0.0241 (5)	−0.0088 (5)	0.0127 (5)	−0.0059 (4)
C63	0.0433 (7)	0.0381 (7)	0.0313 (6)	−0.0210 (6)	0.0234 (6)	−0.0132 (5)
C64	0.0390 (7)	0.0655 (10)	0.0244 (6)	−0.0332 (7)	0.0183 (6)	−0.0187 (6)
C65	0.0200 (5)	0.0824 (11)	0.0143 (5)	−0.0175 (6)	0.0057 (4)	−0.0051 (6)
C66	0.0180 (5)	0.0497 (7)	0.0139 (5)	−0.0051 (5)	0.0073 (4)	0.0006 (5)
C67	0.0148 (4)	0.0225 (5)	0.0131 (4)	0.0003 (4)	0.0036 (4)	−0.0002 (4)
C68	0.0149 (4)	0.0212 (5)	0.0180 (4)	0.0002 (4)	0.0054 (4)	0.0007 (4)
C69	0.0182 (5)	0.0239 (5)	0.0190 (5)	−0.0037 (4)	0.0042 (4)	0.0020 (4)
C70	0.0228 (5)	0.0201 (5)	0.0196 (5)	0.0001 (4)	0.0001 (4)	0.0022 (4)
C71	0.0217 (5)	0.0266 (5)	0.0202 (5)	0.0077 (4)	0.0026 (4)	−0.0009 (4)
C72	0.0192 (5)	0.0303 (6)	0.0170 (5)	0.0059 (4)	0.0064 (4)	0.0017 (4)

### Geometric parameters (Å, º)

**Table:**

N1—C1	1.3407 (13)	C31—C32	1.4021 (13)
N1—C2	1.4639 (13)	C31—C36	1.4073 (13)
N1—C3	1.4644 (13)	C32—C33	1.3966 (14)
N2—C1	1.3364 (13)	C32—H32	0.9500
N2—C5	1.4648 (14)	C33—C34	1.3890 (15)
N2—C4	1.4673 (14)	C33—H33	0.9500
N3—C1	1.3539 (13)	C34—C35	1.3941 (15)
N3—C13	1.4676 (12)	C34—H34	0.9500
N3—C6	1.4820 (12)	C35—C36	1.3912 (14)
N4—C16	1.4994 (13)	C35—H35	0.9500
N4—C17	1.5017 (12)	C36—H36	0.9500
N4—C15	1.5144 (13)	C37—C38	1.4041 (14)
N4—C18	1.5293 (13)	C37—C42	1.4073 (14)
C2—H2A	0.9800	C38—C39	1.3999 (14)
C2—H2B	0.9800	C38—H38	0.9500
C2—H2C	0.9800	C39—C40	1.3862 (17)
C3—H3A	0.9800	C39—H39	0.9500
C3—H3B	0.9800	C40—C41	1.3906 (17)
C3—H3C	0.9800	C40—H40	0.9500
C4—H4A	0.9800	C41—C42	1.3939 (14)
C4—H4B	0.9800	C41—H41	0.9500
C4—H4C	0.9800	C42—H42	0.9500
C5—H5A	0.9800	C43—C48	1.4083 (13)
C5—H5B	0.9800	C43—C44	1.4091 (14)
C5—H5C	0.9800	C44—C45	1.3921 (15)
C6—C7	1.5119 (15)	C44—H44	0.9500
C6—H6A	0.9900	C45—C46	1.3867 (17)
C6—H6B	0.9900	C45—H45	0.9500
C7—C8	1.3963 (15)	C46—C47	1.3882 (17)
C7—C12	1.3965 (15)	C46—H46	0.9500
C8—C9	1.3919 (17)	C47—C48	1.3932 (15)
C8—H8	0.9500	C47—H47	0.9500
C9—C10	1.3823 (18)	C48—H48	0.9500
C9—H9	0.9500	B2—C49	1.6463 (14)
C10—C11	1.3910 (17)	B2—C55	1.6477 (15)
C10—H10	0.9500	B2—C61	1.6512 (16)
C11—C12	1.3895 (17)	B2—C67	1.6521 (15)
C11—H11	0.9500	C49—C54	1.4047 (14)
C12—H12	0.9500	C49—C50	1.4051 (14)
C13—C14	1.5315 (14)	C50—C51	1.3953 (14)
C13—H13A	0.9900	C50—H50	0.9500
C13—H13B	0.9900	C51—C52	1.3904 (15)
C14—C15	1.5205 (13)	C51—H51	0.9500
C14—H14A	0.9900	C52—C53	1.3896 (16)
C14—H14B	0.9900	C52—H52	0.9500
C15—H15A	0.9900	C53—C54	1.3966 (14)
C15—H15B	0.9900	C53—H53	0.9500
C16—H16A	0.9800	C54—H54	0.9500
C16—H16B	0.9800	C55—C56	1.4008 (14)
C16—H16C	0.9800	C55—C60	1.4127 (13)
C17—H17A	0.9800	C56—C57	1.3967 (14)
C17—H17B	0.9800	C56—H56	0.9500
C17—H17C	0.9800	C57—C58	1.3907 (15)
C18—C19	1.5030 (14)	C57—H57	0.9500
C18—H18A	0.9900	C58—C59	1.3897 (16)
C18—H18B	0.9900	C58—H58	0.9500
C19—C24	1.3946 (14)	C59—C60	1.3913 (14)
C19—C20	1.3958 (14)	C59—H59	0.9500
C20—C21	1.3919 (16)	C60—H60	0.9500
C20—H20	0.9500	C61—C66	1.4008 (15)
C21—C22	1.3852 (18)	C61—C62	1.4099 (17)
C21—H21	0.9500	C62—C63	1.3967 (16)
C22—C23	1.3858 (17)	C62—H62	0.9500
C22—H22	0.9500	C63—C64	1.381 (2)
C23—C24	1.3870 (15)	C63—H63	0.9500
C23—H23	0.9500	C64—C65	1.385 (2)
C24—H24	0.9500	C64—H64	0.9500
B1—C43	1.6430 (14)	C65—C66	1.3995 (18)
B1—C31	1.6447 (14)	C65—H65	0.9500
B1—C37	1.6465 (14)	C66—H66	0.9500
B1—C25	1.6505 (15)	C67—C72	1.4080 (14)
C25—C26	1.4040 (14)	C67—C68	1.4082 (13)
C25—C30	1.4075 (14)	C68—C69	1.3991 (15)
C26—C27	1.3979 (15)	C68—H68	0.9500
C26—H26	0.9500	C69—C70	1.3880 (16)
C27—C28	1.3867 (16)	C69—H69	0.9500
C27—H27	0.9500	C70—C71	1.3908 (16)
C28—C29	1.3919 (15)	C70—H70	0.9500
C28—H28	0.9500	C71—C72	1.3917 (16)
C29—C30	1.3885 (15)	C71—H71	0.9500
C29—H29	0.9500	C72—H72	0.9500
C30—H30	0.9500		
			
C1—N1—C2	121.72 (9)	C30—C29—C28	120.04 (10)
C1—N1—C3	123.01 (9)	C30—C29—H29	120.0
C2—N1—C3	115.22 (8)	C28—C29—H29	120.0
C1—N2—C5	122.30 (9)	C29—C30—C25	123.21 (10)
C1—N2—C4	121.50 (9)	C29—C30—H30	118.4
C5—N2—C4	116.13 (9)	C25—C30—H30	118.4
C1—N3—C13	120.42 (8)	C32—C31—C36	115.19 (9)
C1—N3—C6	121.80 (8)	C32—C31—B1	124.25 (8)
C13—N3—C6	117.78 (8)	C36—C31—B1	120.41 (8)
C16—N4—C17	107.85 (8)	C33—C32—C31	122.61 (9)
C16—N4—C15	111.56 (8)	C33—C32—H32	118.7
C17—N4—C15	107.40 (7)	C31—C32—H32	118.7
C16—N4—C18	107.36 (8)	C34—C33—C32	120.42 (9)
C17—N4—C18	110.02 (8)	C34—C33—H33	119.8
C15—N4—C18	112.57 (7)	C32—C33—H33	119.8
N2—C1—N1	121.16 (9)	C33—C34—C35	118.74 (9)
N2—C1—N3	119.26 (9)	C33—C34—H34	120.6
N1—C1—N3	119.57 (9)	C35—C34—H34	120.6
N1—C2—H2A	109.5	C36—C35—C34	119.90 (9)
N1—C2—H2B	109.5	C36—C35—H35	120.1
H2A—C2—H2B	109.5	C34—C35—H35	120.1
N1—C2—H2C	109.5	C35—C36—C31	123.13 (9)
H2A—C2—H2C	109.5	C35—C36—H36	118.4
H2B—C2—H2C	109.5	C31—C36—H36	118.4
N1—C3—H3A	109.5	C38—C37—C42	115.16 (9)
N1—C3—H3B	109.5	C38—C37—B1	124.42 (8)
H3A—C3—H3B	109.5	C42—C37—B1	120.08 (8)
N1—C3—H3C	109.5	C39—C38—C37	122.54 (10)
H3A—C3—H3C	109.5	C39—C38—H38	118.7
H3B—C3—H3C	109.5	C37—C38—H38	118.7
N2—C4—H4A	109.5	C40—C39—C38	120.34 (10)
N2—C4—H4B	109.5	C40—C39—H39	119.8
H4A—C4—H4B	109.5	C38—C39—H39	119.8
N2—C4—H4C	109.5	C39—C40—C41	118.96 (10)
H4A—C4—H4C	109.5	C39—C40—H40	120.5
H4B—C4—H4C	109.5	C41—C40—H40	120.5
N2—C5—H5A	109.5	C40—C41—C42	119.91 (10)
N2—C5—H5B	109.5	C40—C41—H41	120.0
H5A—C5—H5B	109.5	C42—C41—H41	120.0
N2—C5—H5C	109.5	C41—C42—C37	123.07 (10)
H5A—C5—H5C	109.5	C41—C42—H42	118.5
H5B—C5—H5C	109.5	C37—C42—H42	118.5
N3—C6—C7	109.57 (8)	C48—C43—C44	114.87 (9)
N3—C6—H6A	109.8	C48—C43—B1	121.75 (9)
C7—C6—H6A	109.8	C44—C43—B1	123.24 (8)
N3—C6—H6B	109.8	C45—C44—C43	122.86 (10)
C7—C6—H6B	109.8	C45—C44—H44	118.6
H6A—C6—H6B	108.2	C43—C44—H44	118.6
C8—C7—C12	118.81 (10)	C46—C45—C44	120.28 (10)
C8—C7—C6	120.56 (10)	C46—C45—H45	119.9
C12—C7—C6	120.60 (10)	C44—C45—H45	119.9
C9—C8—C7	120.37 (11)	C45—C46—C47	118.82 (10)
C9—C8—H8	119.8	C45—C46—H46	120.6
C7—C8—H8	119.8	C47—C46—H46	120.6
C10—C9—C8	120.20 (11)	C46—C47—C48	120.28 (10)
C10—C9—H9	119.9	C46—C47—H47	119.9
C8—C9—H9	119.9	C48—C47—H47	119.9
C9—C10—C11	120.11 (11)	C47—C48—C43	122.77 (10)
C9—C10—H10	119.9	C47—C48—H48	118.6
C11—C10—H10	119.9	C43—C48—H48	118.6
C12—C11—C10	119.73 (11)	C49—B2—C55	108.64 (8)
C12—C11—H11	120.1	C49—B2—C61	108.73 (8)
C10—C11—H11	120.1	C55—B2—C61	108.51 (8)
C11—C12—C7	120.75 (10)	C49—B2—C67	109.06 (8)
C11—C12—H12	119.6	C55—B2—C67	109.66 (8)
C7—C12—H12	119.6	C61—B2—C67	112.18 (8)
N3—C13—C14	110.54 (8)	C54—C49—C50	115.39 (9)
N3—C13—H13A	109.5	C54—C49—B2	122.73 (9)
C14—C13—H13A	109.5	C50—C49—B2	121.53 (9)
N3—C13—H13B	109.5	C51—C50—C49	122.97 (9)
C14—C13—H13B	109.5	C51—C50—H50	118.5
H13A—C13—H13B	108.1	C49—C50—H50	118.5
C15—C14—C13	108.91 (8)	C52—C51—C50	119.77 (10)
C15—C14—H14A	109.9	C52—C51—H51	120.1
C13—C14—H14A	109.9	C50—C51—H51	120.1
C15—C14—H14B	109.9	C53—C52—C51	119.11 (10)
C13—C14—H14B	109.9	C53—C52—H52	120.4
H14A—C14—H14B	108.3	C51—C52—H52	120.4
N4—C15—C14	114.25 (8)	C52—C53—C54	120.21 (10)
N4—C15—H15A	108.7	C52—C53—H53	119.9
C14—C15—H15A	108.7	C54—C53—H53	119.9
N4—C15—H15B	108.7	C53—C54—C49	122.50 (10)
C14—C15—H15B	108.7	C53—C54—H54	118.7
H15A—C15—H15B	107.6	C49—C54—H54	118.7
N4—C16—H16A	109.5	C56—C55—C60	115.27 (9)
N4—C16—H16B	109.5	C56—C55—B2	123.89 (8)
H16A—C16—H16B	109.5	C60—C55—B2	120.83 (9)
N4—C16—H16C	109.5	C57—C56—C55	122.51 (9)
H16A—C16—H16C	109.5	C57—C56—H56	118.7
H16B—C16—H16C	109.5	C55—C56—H56	118.7
N4—C17—H17A	109.5	C58—C57—C56	120.37 (10)
N4—C17—H17B	109.5	C58—C57—H57	119.8
H17A—C17—H17B	109.5	C56—C57—H57	119.8
N4—C17—H17C	109.5	C59—C58—C57	118.96 (9)
H17A—C17—H17C	109.5	C59—C58—H58	120.5
H17B—C17—H17C	109.5	C57—C58—H58	120.5
C19—C18—N4	114.89 (8)	C58—C59—C60	119.90 (9)
C19—C18—H18A	108.5	C58—C59—H59	120.1
N4—C18—H18A	108.5	C60—C59—H59	120.1
C19—C18—H18B	108.5	C59—C60—C55	122.97 (10)
N4—C18—H18B	108.5	C59—C60—H60	118.5
H18A—C18—H18B	107.5	C55—C60—H60	118.5
C24—C19—C20	119.33 (10)	C66—C61—C62	115.02 (10)
C24—C19—C18	119.01 (9)	C66—C61—B2	124.16 (10)
C20—C19—C18	121.59 (9)	C62—C61—B2	120.72 (9)
C21—C20—C19	119.76 (10)	C63—C62—C61	123.07 (12)
C21—C20—H20	120.1	C63—C62—H62	118.5
C19—C20—H20	120.1	C61—C62—H62	118.5
C22—C21—C20	120.27 (11)	C64—C63—C62	120.12 (14)
C22—C21—H21	119.9	C64—C63—H63	119.9
C20—C21—H21	119.9	C62—C63—H63	119.9
C21—C22—C23	120.33 (11)	C63—C64—C65	118.60 (12)
C21—C22—H22	119.8	C63—C64—H64	120.7
C23—C22—H22	119.8	C65—C64—H64	120.7
C22—C23—C24	119.58 (11)	C64—C65—C66	120.96 (13)
C22—C23—H23	120.2	C64—C65—H65	119.5
C24—C23—H23	120.2	C66—C65—H65	119.5
C23—C24—C19	120.71 (10)	C65—C66—C61	122.22 (13)
C23—C24—H24	119.6	C65—C66—H66	118.9
C19—C24—H24	119.6	C61—C66—H66	118.9
C43—B1—C31	104.53 (7)	C72—C67—C68	114.73 (9)
C43—B1—C37	111.84 (8)	C72—C67—B2	120.29 (9)
C31—B1—C37	112.32 (8)	C68—C67—B2	124.92 (9)
C43—B1—C25	113.09 (8)	C69—C68—C67	122.66 (10)
C31—B1—C25	110.61 (8)	C69—C68—H68	118.7
C37—B1—C25	104.66 (7)	C67—C68—H68	118.7
C26—C25—C30	114.94 (9)	C70—C69—C68	120.56 (10)
C26—C25—B1	126.45 (9)	C70—C69—H69	119.7
C30—C25—B1	118.57 (8)	C68—C69—H69	119.7
C27—C26—C25	122.68 (10)	C69—C70—C71	118.51 (10)
C27—C26—H26	118.7	C69—C70—H70	120.7
C25—C26—H26	118.7	C71—C70—H70	120.7
C28—C27—C26	120.33 (10)	C70—C71—C72	120.24 (10)
C28—C27—H27	119.8	C70—C71—H71	119.9
C26—C27—H27	119.8	C72—C71—H71	119.9
C27—C28—C29	118.77 (10)	C71—C72—C67	123.29 (10)
C27—C28—H28	120.6	C71—C72—H72	118.4
C29—C28—H28	120.6	C67—C72—H72	118.4
			
C5—N2—C1—N1	30.14 (15)	C42—C37—C38—C39	1.15 (15)
C4—N2—C1—N1	−146.67 (10)	B1—C37—C38—C39	−172.02 (9)
C5—N2—C1—N3	−150.09 (10)	C37—C38—C39—C40	−0.37 (16)
C4—N2—C1—N3	33.10 (14)	C38—C39—C40—C41	−0.70 (16)
C2—N1—C1—N2	−147.59 (10)	C39—C40—C41—C42	0.93 (16)
C3—N1—C1—N2	34.77 (14)	C40—C41—C42—C37	−0.10 (17)
C2—N1—C1—N3	32.64 (14)	C38—C37—C42—C41	−0.92 (15)
C3—N1—C1—N3	−145.00 (10)	B1—C37—C42—C41	172.57 (10)
C13—N3—C1—N2	−136.31 (10)	C31—B1—C43—C48	74.29 (11)
C6—N3—C1—N2	44.00 (14)	C37—B1—C43—C48	−163.94 (9)
C13—N3—C1—N1	43.47 (13)	C25—B1—C43—C48	−46.09 (12)
C6—N3—C1—N1	−136.23 (10)	C31—B1—C43—C44	−101.32 (10)
C1—N3—C6—C7	124.68 (10)	C37—B1—C43—C44	20.44 (13)
C13—N3—C6—C7	−55.02 (11)	C25—B1—C43—C44	138.30 (9)
N3—C6—C7—C8	107.17 (10)	C48—C43—C44—C45	2.20 (15)
N3—C6—C7—C12	−70.71 (12)	B1—C43—C44—C45	178.09 (10)
C12—C7—C8—C9	1.94 (15)	C43—C44—C45—C46	0.80 (17)
C6—C7—C8—C9	−175.97 (9)	C44—C45—C46—C47	−2.22 (17)
C7—C8—C9—C10	−1.02 (16)	C45—C46—C47—C48	0.54 (17)
C8—C9—C10—C11	−0.36 (17)	C46—C47—C48—C43	2.69 (17)
C9—C10—C11—C12	0.80 (17)	C44—C43—C48—C47	−3.93 (15)
C10—C11—C12—C7	0.15 (16)	B1—C43—C48—C47	−179.89 (9)
C8—C7—C12—C11	−1.51 (15)	C55—B2—C49—C54	−90.58 (11)
C6—C7—C12—C11	176.41 (9)	C61—B2—C49—C54	27.32 (13)
C1—N3—C13—C14	119.40 (10)	C67—B2—C49—C54	149.93 (9)
C6—N3—C13—C14	−60.90 (11)	C55—B2—C49—C50	82.32 (11)
N3—C13—C14—C15	−163.36 (8)	C61—B2—C49—C50	−159.77 (9)
C16—N4—C15—C14	−46.69 (11)	C67—B2—C49—C50	−37.17 (12)
C17—N4—C15—C14	−164.67 (8)	C54—C49—C50—C51	2.28 (15)
C18—N4—C15—C14	74.08 (10)	B2—C49—C50—C51	−171.12 (9)
C13—C14—C15—N4	−171.78 (8)	C49—C50—C51—C52	−1.13 (16)
C16—N4—C18—C19	177.68 (8)	C50—C51—C52—C53	−0.67 (16)
C17—N4—C18—C19	−65.21 (11)	C51—C52—C53—C54	1.16 (16)
C15—N4—C18—C19	54.53 (11)	C52—C53—C54—C49	0.11 (16)
N4—C18—C19—C24	−99.44 (11)	C50—C49—C54—C53	−1.76 (15)
N4—C18—C19—C20	83.67 (12)	B2—C49—C54—C53	171.54 (10)
C24—C19—C20—C21	1.63 (16)	C49—B2—C55—C56	−7.72 (13)
C18—C19—C20—C21	178.51 (10)	C61—B2—C55—C56	−125.77 (10)
C19—C20—C21—C22	−1.29 (18)	C67—B2—C55—C56	111.39 (10)
C20—C21—C22—C23	0.03 (19)	C49—B2—C55—C60	173.08 (9)
C21—C22—C23—C24	0.87 (18)	C61—B2—C55—C60	55.03 (11)
C22—C23—C24—C19	−0.51 (16)	C67—B2—C55—C60	−67.81 (11)
C20—C19—C24—C23	−0.74 (15)	C60—C55—C56—C57	1.07 (14)
C18—C19—C24—C23	−177.69 (9)	B2—C55—C56—C57	−178.18 (9)
C43—B1—C25—C26	−4.50 (13)	C55—C56—C57—C58	0.11 (16)
C31—B1—C25—C26	−121.35 (10)	C56—C57—C58—C59	−0.67 (15)
C37—B1—C25—C26	117.47 (10)	C57—C58—C59—C60	−0.02 (15)
C43—B1—C25—C30	177.93 (8)	C58—C59—C60—C55	1.31 (15)
C31—B1—C25—C30	61.08 (11)	C56—C55—C60—C59	−1.79 (14)
C37—B1—C25—C30	−60.10 (10)	B2—C55—C60—C59	177.48 (9)
C30—C25—C26—C27	−2.03 (14)	C49—B2—C61—C66	90.18 (11)
B1—C25—C26—C27	−179.67 (9)	C55—B2—C61—C66	−151.82 (9)
C25—C26—C27—C28	1.11 (16)	C67—B2—C61—C66	−30.51 (13)
C26—C27—C28—C29	0.66 (16)	C49—B2—C61—C62	−86.04 (11)
C27—C28—C29—C30	−1.36 (16)	C55—B2—C61—C62	31.96 (12)
C28—C29—C30—C25	0.36 (16)	C67—B2—C61—C62	153.26 (9)
C26—C25—C30—C29	1.30 (14)	C66—C61—C62—C63	0.73 (15)
B1—C25—C30—C29	179.14 (9)	B2—C61—C62—C63	177.28 (10)
C43—B1—C31—C32	−102.65 (10)	C61—C62—C63—C64	0.46 (18)
C37—B1—C31—C32	135.90 (9)	C62—C63—C64—C65	−0.78 (18)
C25—B1—C31—C32	19.38 (12)	C63—C64—C65—C66	−0.10 (18)
C43—B1—C31—C36	72.61 (10)	C64—C65—C66—C61	1.38 (17)
C37—B1—C31—C36	−48.84 (12)	C62—C61—C66—C65	−1.63 (15)
C25—B1—C31—C36	−165.37 (8)	B2—C61—C66—C65	−178.05 (9)
C36—C31—C32—C33	1.25 (14)	C49—B2—C67—C72	−46.45 (12)
B1—C31—C32—C33	176.73 (9)	C55—B2—C67—C72	−165.31 (9)
C31—C32—C33—C34	−0.23 (15)	C61—B2—C67—C72	74.05 (12)
C32—C33—C34—C35	−0.87 (15)	C49—B2—C67—C68	130.67 (10)
C33—C34—C35—C36	0.85 (15)	C55—B2—C67—C68	11.82 (13)
C34—C35—C36—C31	0.25 (15)	C61—B2—C67—C68	−108.82 (11)
C32—C31—C36—C35	−1.27 (14)	C72—C67—C68—C69	−0.65 (15)
B1—C31—C36—C35	−176.94 (9)	B2—C67—C68—C69	−177.92 (10)
C43—B1—C37—C38	−137.32 (10)	C67—C68—C69—C70	0.54 (16)
C31—B1—C37—C38	−20.16 (13)	C68—C69—C70—C71	−0.02 (16)
C25—B1—C37—C38	99.88 (10)	C69—C70—C71—C72	−0.34 (16)
C43—B1—C37—C42	49.82 (12)	C70—C71—C72—C67	0.20 (17)
C31—B1—C37—C42	166.99 (9)	C68—C67—C72—C71	0.29 (16)
C25—B1—C37—C42	−72.97 (11)	B2—C67—C72—C71	177.70 (10)

### Hydrogen-bond geometry (Å, º)

Cg1, Cg2 and Cg3 are the centroids of the C31–C36, C43–C48 and C61–C66 aromatic rings, respectively.

**Table:**

*D*—H···*A*	*D*—H	H···*A*	*D*···*A*	*D*—H···*A*
C2—H2*B*···*Cg*1^i^	0.98	2.76	3.441	127
C15—H15*A*···*Cg*1^ii^	0.99	2.94	3.538	120
C17—H17*A*···*Cg*2^ii^	0.98	2.76	3.716	166
C4—H4*A*···*Cg*3^iii^	0.98	2.88	3.840	169
C20—H20···*Cg*3^iv^	0.95	2.91	3.719	144

Symmetry codes: (i) −*x*+1, *y*+1/2, −*z*+1/2; (ii) *x*, −*y*+1/2, *z*−1/2; (iii) −*x*, *y*−1/2, −*z*+1/2; (iv) −*x*, −*y*+1, −*z*.
