# Supplementary figures and images for: Crystal structure of N′′-benzyl-N′′-[3-(benzyl­dimethyl­aza­nium­yl)prop­yl]-N,N,N′,N′-tetra­methyl­guanidinium bis­(tetra­phenyl­borate)
Source: Acta Crystallogr E Crystallogr Commun. 2015 Dec 31;71(Pt 12):o1086–7. doi: 10.1107/S2056989015024639 (PMC4719992; doi:10.1107/S2056989015024639)

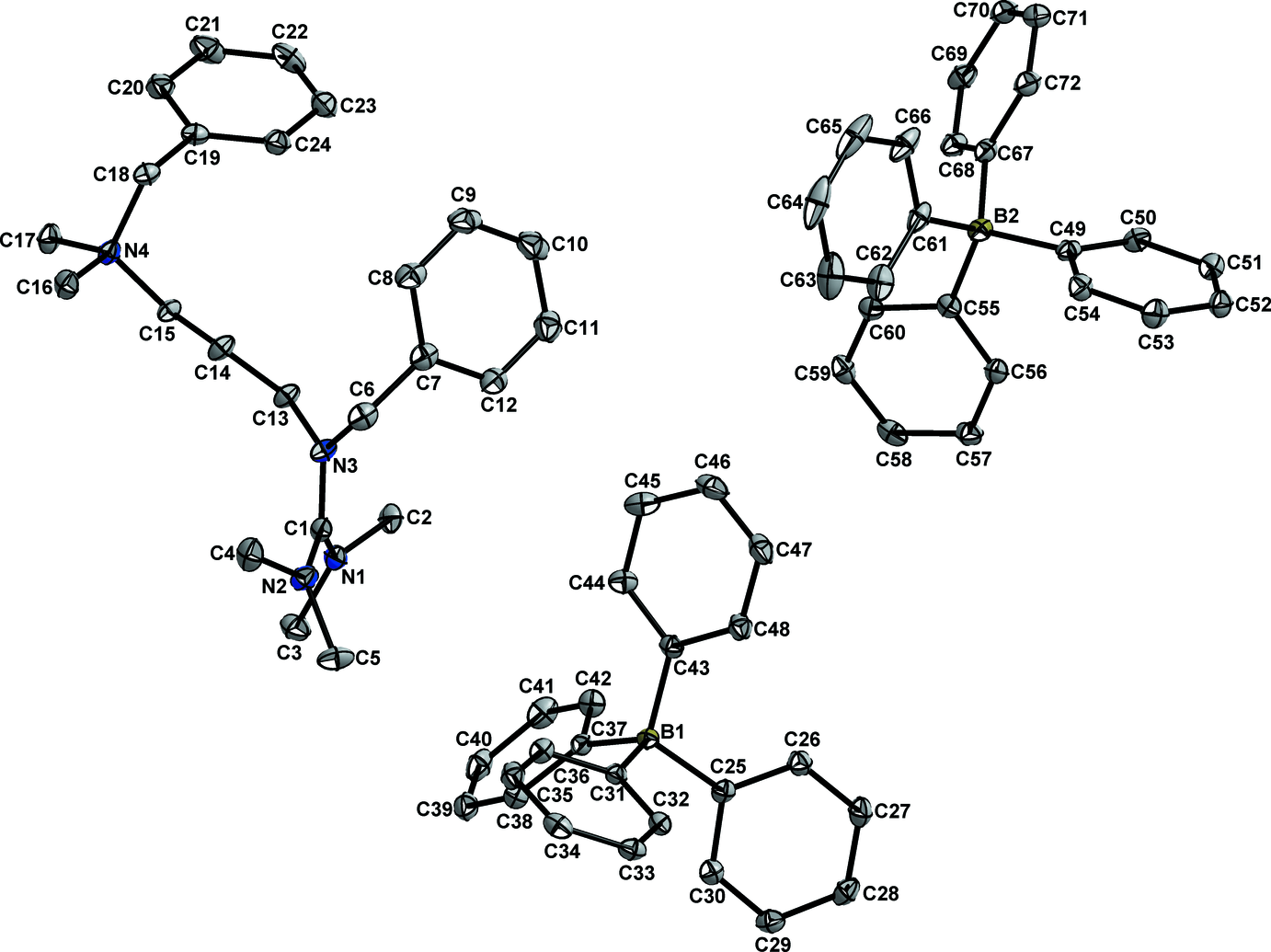

Supplement: Supplementary file 4 [file e-71-o1086-fig1.tif]

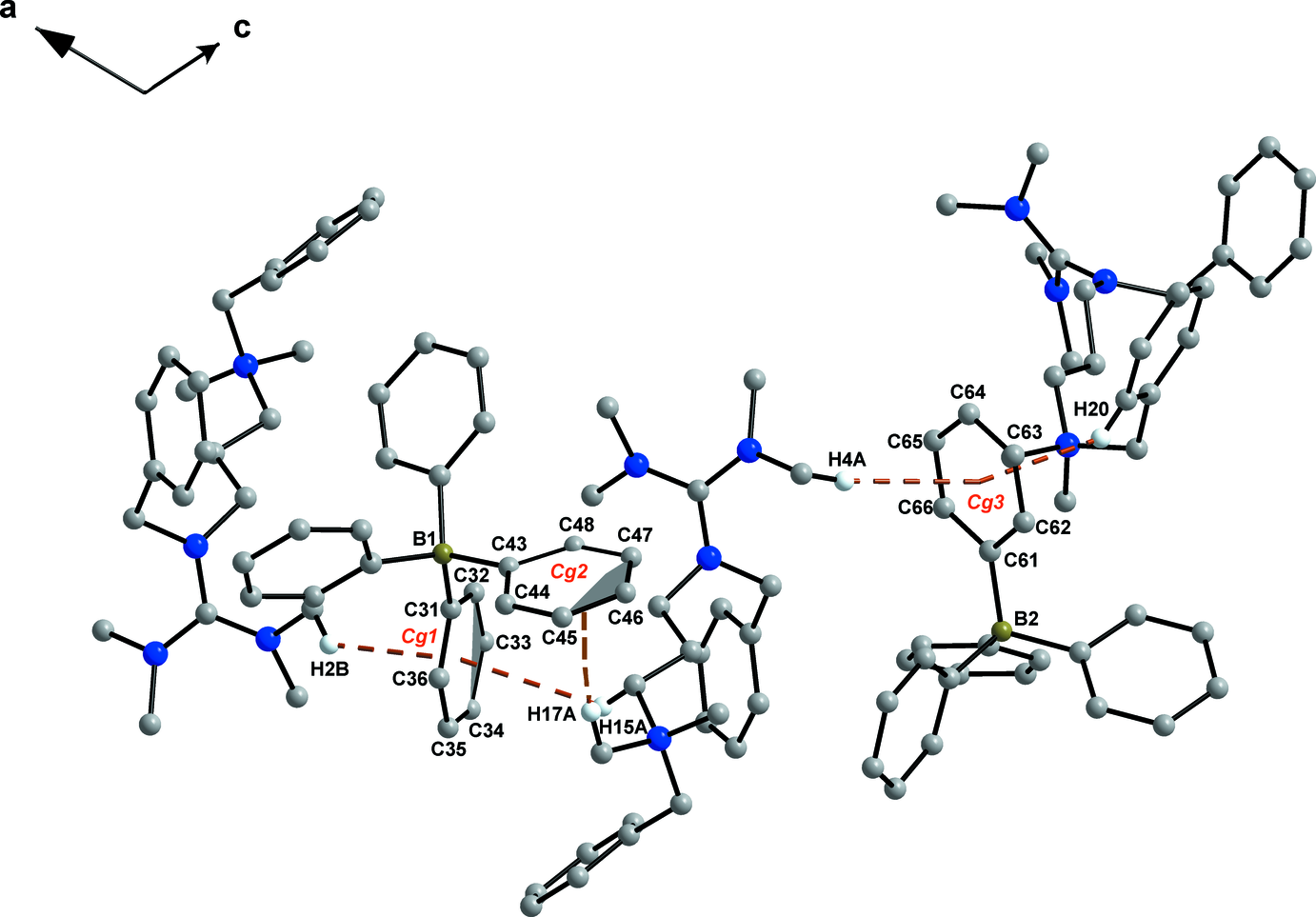

Supplement: Supplementary file 5 [file e-71-o1086-fig2.tif]
